# The Combination of Blueberry Juice and Probiotics Ameliorate Non-Alcoholic Steatohepatitis (NASH) by Affecting SREBP-1c/PNPLA-3 Pathway via PPAR-α

**DOI:** 10.3390/nu9030198

**Published:** 2017-02-27

**Authors:** Tingting Ren, Juanjuan Zhu, Lili Zhu, Mingliang Cheng

**Affiliations:** 1 Biochemistry Department, Affiliated Hospital of Guiyang Medical College, Guiyang 550004, China; tingting1@163.com; 2 Department of Infectious Diseases, Affiliated Hospital of Guiyang Medical College, Guiyang 550004, China; juanjuan2@163.com; 3 Baiyun Hospital, Affiliated Hospital of Guiyang Medical College, Guiyang 550004, China; zhulili1@163.com

**Keywords:** non-alcoholic steatohepatitis, blueberry juices, probiotics, Peroxisome proliferator-activated receptor α, Sterol regulatory element-binding transcription factor 1c, Patatin-like phospholipase domain-containing protein 3, biochemical indices, apoptosis, anti-oxidant

## Abstract

Nonalcoholic steatohepatitis (NASH) is liver inflammation and a major threat to public health. Several pharmaceutical agents have been used for NASH therapy but their high-rate side effects limit the use. Blueberry juice and probiotics (BP) have anti-inflammation and antibacterial properties, and may be potential candidates for NASH therapy. To understand the molecular mechanism, Sprague Dawley rats were used to create NASH models and received different treatments. Liver tissues were examined using HE (hematoxylin and eosin) and ORO (Oil Red O) stain, and serum biochemical indices were measured. The levels of peroxisome proliferators-activated receptor (PPAR)-α, sterol regulatory element binding protein-1c (SREBP-1c), Patatin-like phospholipase domain-containing protein 3 (PNPLA-3), inflammatory cytokines and apoptosis biomarkers in liver tissues were measured by qRT-PCR and Western blot. HE and ORO analysis indicated that the hepatocytes were seriously damaged with more and larger lipid droplets in NASH models while BP reduced the number and size of lipid droplets (*p* < 0.05). Meanwhile, BP increased the levels of SOD (superoxide dismutase), GSH (reduced glutathione) and HDL-C (high-density lipoprotein cholesterol), and reduced the levels of AST (aspartate aminotransferase), ALT (alanine aminotransferase), TG (triglycerides), LDL-C (low-density lipoprotein cholesterol) and MDA (malondialdehyde) in NASH models (*p* < 0.05). BP increased the level of PPAR-α (Peroxisome proliferator-activated receptor α), and reduced the levels of SREBP-1c (sterol regulatory element binding protein-1c) and PNPLA-3 (Patatin-like phospholipase domain-containing protein 3) (*p* < 0.05). BP reduced hepatic inflammation and apoptosis by affecting IL-6 (interleukin 6), TNF-α (Tumor necrosis factor α), caspase-3 and Bcl-2 in NASH models. Furthermore, PPAR-α inhibitor increased the level of SREBP-1c and PNPLA-3. Therefore, BP prevents NASH progression by affecting SREBP-1c/PNPLA-3 pathway via PPAR-α.

## 1. Introduction

Non-alcoholic steatohepatitis (NASH) is a kind of liver diseases caused by oxidant and inflammation stress over a long time and results in liver damage. NASH can cause some serious complications, such as liver failure [1], cirrhosis [2,3] and hepatocellular carcinoma [4]. Furthermore, NASH risk is increasing worldwide [5,6,7]. Drug therapy is still the main option to control NASH progression [8,9,10]. The common drugs for NASH treatment are vitamin E [11], pioglitazone [12], peroxisome proliferator-activated receptors (PPAR)-α and PPAR-γ agonist [13], etc. Vitamin E has antioxidant activities and is widely used for treating chronic liver disorders. Vitamin E lowers the levels of alanine aminotransferase (ALT) and aspartate aminotransferase (AST) in patients with NASH [14]. Pioglitazone shows inhibitory effects on the level of vascular endothelial growth factor (VEGF), which is related to many ischemic and inflammatory disorders, and the main factor contributing to the progression of liver fibrosis and hepatic carcinogenesis [15]. The activation of PPAR-α and PPAR-γ ameliorates NASH by regulating the gene expression in hepatic and adipose tissues [13]. However, all these medicines have obvious side effects. Long-term consumption of vitamin E will cause nausea, vomiting, diarrhea, headache, dizziness, and cerebrovascular disorders, as well as increase the risk of cancers [16]. Pioglitazone belongs to PPAR-γ agonist thiazolidinedione and has many side effects [17], including weight gain, pedal edema, bone loss and heart failure [18]. Thiazolidinedione, as an extracellular signal-regulated kinase (ERK) docking domain inhibitor, may cause angioneurosis edema. PPAR-γ, an important regulator of lipid metabolism and energy balance, is involved in the progression of insulin resistance and obesity [19]. Furthermore, thiazolidinedione may induce side effects via PPAR since thiazolidinedione-induced activation of PPAR-γ changes the transcription of many genes associated with glucose and lipid metabolism [20]. Generally, these side effects cannot be tolerated by most patients. Thus, it is critical to find non-pharmaceutical therapy and fruit-based food products for NASH treatment with few side effects.

Oxidative stress may be an important factor for causing NASH. Genetic SNPs (single nucleotide polymorphisms) have been reported to be associated with fatty acid oxidation and contribute to NASH [21]. An earlier report showed that increasing CYP2E1 (Catalase and cytochrome P450 2E1) will increase oxidative stress and plays an important role in the progression of NASH [22]. Some data suggested that NASH patients could receive antioxidant therapies according to the oxidative stresses in their serum [23]. Inflammation is another important factor causing NASH because NASH is mainly characterized by hepatic lipid accumulation and is associated with progressive inflammatory liver disease [24,25]. The liver kinetics of gadolinium-ethoxybenzyl-diethylenetriamine-pentaacetic acid and complications are associated with inflammation in animal NASH models [26]. The distribution of gut microbiota also plays an important role in the progression of NASH [27].

Blueberry juice has significant anti-oxidant and anti-inflammation functions. Blueberry has rich anthocyanins, which protect organs from oxidative destruction and it can be used as functional fruits to control liver disorders caused by oxidative stress [28]. The anthocyanins can decrease the levels of reactive oxygen species (ROS) and heme oxygenase-1 (HO-1) and increase the levels of superoxide dismutase (SOD) and HO-1. Anthocyanins show protecting effects for inflammatory disorders and protect cells from diabetes-induced oxidant and inflammatory injuries by affecting Nrf-2/HO-1 (Nuclear factor erythroid 2-related factor 2/heme oxygenase-1) signal pathway [29]. The rich glycosides in blueberries also enhance their antioxidant capacities [28]. Phenolic acid is also an important composition of blueberry and antioxidant protection for most cell and. The mixture of phenolic acid in blueberry also shows anti-inflammatory functions by reducing the levels of nuclear factor-kappa B and increasing the level of tumor necrosis factor (TNF)-α and interleukin-6 (IL-6) [30]. Blueberry has abundant polyphenols and some beneficial compounds, which can be used by gut probiotics and affect digestive system and the distribution of intestinal microflora. Bifidobacterium, a kind of probiotics, is considered beneficial for human health. Long-term consumption of blueberry juice has been proven to improve the distribution of the intestinal microflora [31]. On the other hand, blueberries provide health benefits for preventing the progression of various cancers. The anticancer functions of blueberries are attributed to their high-content phytochemicals and antioxidant properties. The main ingredients of blueberries show protective effects against cancer by suppressing inflammation, oxidative stress, proliferation and angiogenesis via many pathways including nuclear factor kappa-B, Wnt/beta-catenin, the phosphatidylinositol-3-kinase and the mammalian target of Rapamycin, and extracellular signal-regulated kinase/mitogen-activated protein kinase [32]. Blueberry-enriched diet can control metabolic disorders including endothelial dysfunction and inflammation in the obese animal models [33]. Probiotics have also been approved for NASH treatment in an animal model [34]. Thus, the combination of blueberry juice and probiotics (BP) may have protective functions in NASH therapy since inflammation, oxidant stress and gut microbiota are associated with the development of NASH.

However, the molecular mechanism of the effects of blueberry juice on the development of NASH remains widely unclear. The mutual interaction between lipid metabolism and inflammation may exacerbate atherosclerosis progression [35], which will contribute to NASH [36]. Sterol regulatory element-binding protein-1c (SREBP-1c) and PPAR-α have been reported to affect the development of NASH. Low-level expression of PPAR-α will induce the risk of NASH, which can be treated well by chicory (*Cichoriumintybus* L.) seed extract by affecting the level of PPAR-α [37] while PPAR-α activation will inhibit SREBP-1c pathway [38]. On the other hand, SREBP-1c and patatin-like phospholipase domain-containing protein 3 (PNPLA-3) pathways may affect the progression of NASH [39,40]. The pathway may be associated with inflammation [41] and oxidative stress [42]. PNPLA-3 has been reported to be related to the increase in the level of total glycerol and risk of hepatic injuries [43]. The PNPLA-3 mutant is associated with the risks of hepatic steatosis [44], hepatic enzymes [45], hepatosteatosis [46], and alcohol-induced cirrhosis [47]. PNPLA-3 encodes a 481-aa protein with apatatin-like domain at the *N*-terminus. The over-expression of PNPLA-3 has been found in adipose tissue [48]. PNPLA-3 is similar with PNPLA2, which is a main kind of hormone-sensitive lipase in adipose tissue. Both PNPLA2 and PNPLA-3 can hydrolyze triglyceride [49]. Furthermore, PNPLA-3 level is higher in obese and insulin-resistant animal models [50]. 

Blueberry may affect NASH development by affecting SREBP-1c/PNPLA-3 pathway. Therefore, the molecular mechanism for the functional role of BP in NASH treatment was explored by investigating SREBP-1c/PNPLA-3 pathway. Meanwhile, the modulator PPAR-α of SREBP-1c/PNPLA-3 pathway, and oxidative and inflammatory factors were also measured.

## 2. Materials and Methods

### 2.1. Animals

All the protocols were approved by the animal care and ethical committee of Affiliated Hospital of Guiyang Medical College (Approval No. GY2015R29). A total of 56 male Sprague Dawley rats (6- to 8-week old and 250 ± 20 g) were purchased from the experimental animal center, Third Military Medical University (Chongqing, China). All animal-handling procedures were performed according to the guide for the care and use of laboratory animals of NIH, and followed the guidelines of the animal welfare act. All animals were housed in a 12 Light:12 Dark cycle with ad libitum access to food and water.

### 2.2. Materials

Blueberries were purchased from Majiang Blueberry Plant (Guiyang, China) and preserved at −20 °C immediately. Blueberry juice was prepared according to an earlier report [51]. Briefly, 1 kg blueberry blend was thawed at 4 °C for 8 h and milled using Braun Global Hand Blender MR300 (De’Longhi Kenwood A.P.A Ltd., Hong Kong, China). The crushed blueberries were pressed in a bag press mod with the maximum pressure at 0.9 MPa. The blueberry juice was used as food for rats immediately. The main composition of blueberry juice was measured according to an earlier report [52]. The probiotics mixture with *Bifidobacterium lactis*, *Lactobacillus bulgaricus* and *Streptococcus thermophilus* were from Inner Mongolia Double Odd Pharmaceutical Co. (Tongliao, China). Probiotics were prepared freshly at log stage and used immediately. RNA purification kit was from Thermo Fisher Scientific (Waltham, MA, USA), SYBR Premix Ex Taq and PrimeScript RT reagent Kit from TaKaRa Bio Inc. (Dalian, Liaoning, China). SREBP-1c rabbit anti-rat antibodies (ab28481) and Anti-PNPLA-3 antibody (ab69170) were from Abcam (Cambridge, MA, USA). Monoclonal rabbit anti-rat PPAR-α antibody (sc-50252) was from Santa Cruz Biotechnology Inc. (Delaware Ave., Santa Cruz, CA, USA). Rabbit anti-rat IL-6 antibody (ab6672), rabbit anti-rat TNF (tumor necrosis factor) antibody (ab6671), rabbit anti-rat caspase-3 antibody (ab44976) and rabbit anti-rat BCL-2 antibody (ab59348) were also from Abcam. Caspase 3 GAPDH rabbit anti-rat antibody (ab37168) and Goat anti-rabbit IgG H&L (HRP, ab6721) were from Abcam. 

### 2.3. The Effects of PPAR-α Antagonist and Blueberry Juice on the Growth of Liver Cells

According to previous reports [53,54,55], human liver cell line HL7702 and HepG2 were selected for NASH research and purchased from Shanghai Cell bank (Chinese Academy of Science, Shanghai, China), and cultured in DMEM with 25 mM glucose, 5 mM glutamine, 10% fetal bovine serum (FBS), 100 μg/mL penicillin and 100 μg/mL streptomycin at 37 °C under a 5% CO_2_ environment. The cell concentrations were adjusted to 1 × 10^5^/mL and 200 μL was added to each cell in a 96-cell plate. GW6471, a PPAR-α antagonist, was purchased from Santa Cruz Biotechnology, Inc. (Santa Cruz, CA, USA). Either 20 μg/mL GW6471 or 10 μg/mL blueberry juice, or both, was added to each cell in a 96-cell plate and further cultured for 3 days. Adherent cells were detached by using 0.1% trypsin and 0.04% EDTA and resuspended in CASYton solution (Roche Applied Science, cat. no. 05651808 001). The debris was removed using a 100-μm nylon filter (Falcon Business USA Inc., Fairfield, CT, USA). Cell concentrations were measured using a glass hemocytometer and coverslip daily.

### 2.4. The Establishment of NASH Rat Model and Animal Groups

Eight Sprague-Dawley rats were fed with a normal diet as a control group (Table 1). Forty-eight Sprague-Dawley rats were fed with high-fat diet (normal diet + two percent cholesterol and ten percent lard) for two months (Table 1). The NASH rat model was evaluated by a histological feature scoring system that addresses the full spectrum of lesions of NAFLD and NASH. A NAFLD activity score (NAS) ≥5 was associated with a diagnosis of NASH, and less than 3 would be diagnosed as “not NASH” [56]. Twenty-four NASH rats were intra-articularly intraperitoneally injected with PPAR-α antagonist, GW6471, in 20 mL/kg solution for a week.

**Figure 1:**
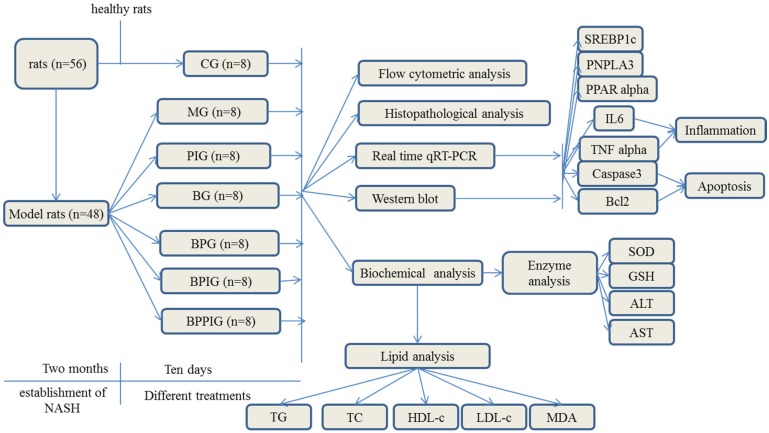
The flow chart of the study.

The doses of blueberry [57], probiotics [58], and PPAR α antagonist (GW6471) [59] were administrated according to previous reports. As shown in Figure 1, all rat NASH models were evenly and randomly assigned to 6 groups: model group (MG, the model rats were intraperitoneally injected with 50 μL/kg saline solution and orally received 20 mL/kg liquid placebo daily); blueberry juice group (BG, the model rats were intraperitoneally injected with 50 μL/kg saline solution and orally received 10 mL/kg blueberry juice and 10 mL/kg liquid placebo daily); Blueberry juice and probiotic bacteria group (BPG, the model rats were intraperitoneally injected with 50 μL/kg normal saline solution and orally received 10 mL/kg blueberry juice and 10 mL/kg probiotics daily); PPAR-α inhibitor group (PIG, the model rats were intraperitoneally injected with 50 μL/kg PPAR-α in saline solution); blueberry juice and PPAR-α inhibitor group (BPIG, the model rats were intraperitoneally injected with 50 μL/kg PPAR-α in saline solution and orally received 10 mL/kg blueberry juice and 10 mL/kg liquid placebo daily); and blueberry juice, probiotics, and PPAR-α inhibitor group (BPPIG, the model rats were intraperitoneally injected with 50 μL/kg PPAR-α in saline solution and orally received 10 mL/kg blueberry juice and 10 mL/kg probiotics daily). Meanwhile, 8 healthy rats were used as a control group (CG, the healthy rats were intraperitoneally injected with 50 μL/kg normal saline solution and orally received 20 mL/kg liquid placebo daily). The whole treatment period was ten days.

### 2.5. Measurement of PPAR-α

The activity of PPAR-α was measured using PPAR-α assay kit (Cat. No. ab133107, Abcam (Shanghai) branch, Shanghai, China) among different groups after ten-day blueberry and/or BP treatment. 

### 2.6. Histological Analysis

After ten-day blueberry and probiotic and/or -PPAR-α treatment, all rats were sacrificed by decapitation. Blood samples were obtained and centrifuged at 5000× *g* for 10 min, and serum was purified and stored at −80 °C. Liver tissue was cut and fixed in ten percent formalin, and paraffin-embedded sections were prepared. All sections were stained by hematoxylin and eosin (HE) and Oil Red O (ORO). The sections were observed under an A12.1503 microscope (Opto-Edu (Beijing) Co., Ltd., Beijing, China). The diameters of droplets were calculated using a hemocytometer. After being stained with HE, the sections were washed with ddH_2_O and 60% isopropanol for three times. Oil Red O was extracted with 100% isopropanol for five minutes. The solution was measured at 540 nm.

### 2.7. Flow Cytometry Analysis

The apoptotic rates of hepatic cells were measured by using an EC800 flow cytometer (Shinagawa-ku, Tokyo, Japan). All hepatic tissues were pulverized using a pestle and mortar and single cells were separated by a Nycodenz density gradient. A total of 100 µL of cells with the concentration of 1 × 10^5^/mL were transferred into 5-mL flow tubes. Annexin V, conjugated to green-fluorescent FITC dye, was added, and apoptotic rate was calculated according to the fluorescence intensity of Annexin V-FITC.

### 2.8. Biochemical Analysis

Some molecules involving in oxidative stress were analyzed because oxidative stress is an important element for the development of NASH [21,22]. The serum activity of superoxide dismutase (SOD) was measured by formazan-WST method [60]. WST1 produces water soluble formazan dye on reduction with superoxide anion. Reduction rate is linearly associated with the activities of anthine oxidase, which can be repressed by SOD. Serum sample was treated with WST for 20 min at 37 °C. The absorbing values were recorded at 450 nm and inhibiting rates were calculated. The term “0.5 O.D.” is defined as having 1 unit of SOD activity. The serum concentration of reduced glutathione (GSH) was determined by the Dithiobis-2-nitrobenzoic acid (DTNB) method [61]. DTNB reacts with the sulfhydryl group of GSH and produces yellow TNB and GS-TNB, which is then reduced by GSH and β-nicotinamide adenine dinucleotide phosphate (β-NADPH). The TNB production is associated with the concentration of GSH in serum samples. TNB absorbing values were measured at 412 nm. One unit is defined as the amount of enzyme required to catalyze the reduction of one micromole of substrate per min. The serum concentrations of aspartate amino-transaminase (AST) and alanine aminotransferase (ALT) were evaluated by using Hitachi 7170A/7180 Biochemical Analyzer (Hitachi, Japan). Malondialdehyde (MDA) serum concentrations were determined by using an MDA kit (GENMED, Shanghai, China). The units for AST and ALT are defined as one absorbing unit per minute in the serum samples. NASH development is often associated with the disorder of lipid metabolism. Therefore, the serum levels of total triglycerides (TG), total cholesterol (TC), high-density lipoprotein-cholesterol (HDL-C), and low-density lipoprotein-cholesterol (LDL-C) were also determined by Hitachi 7170A/7180 Biochemical Analyzer. Serum levels of IL-6 and TNF-α were measured by using different ELISA kits (Cat. No. ab100772 and ab108913) from Abcam (Cambridge, MA, USA).

### 2.9. qRT-PCR

Total hepatic RNA was exacted and purified by using a RNA purification kit. cDNA was produced based on the instructions of RT-PCR kit. qRT-PCR was performed to assay the mRNA levels of PPAR-α SREBP-1c, PNPLA-3, caspase-3 and Bcl-2 genes. GAPDH was a control to normalize the copy number of each sample. All the primer sequences are listed in Table 2. qRT-PCR was performed on The CFX96 Touch Real-Time PCR Detection System (Bio-Rad, Hercules, CA, USA). The mean Ct value represented the mRNA levels of the individual gene.

### 2.10. Western Blot Analysis

Protein was extracted and the concentration was determined by a Bradford protein assay kit (Beyotime Biotechnology, Beijing, China). Thirty micrograms of protein were taken from each group, separated by 12% sodium dodecyl sulfate polyacrylamide gel electrophoresis (SDS-PAGE), and then transferred to a Polyvinylidene difluoride (PVDF) membranes (Millipore Corporation, Bedford, MA, USA), which was blocked by 5% non-fat milk. The membrane was treated with primary antibodies at 4 °C for 10 h. Secondary antibodies were added and incubated for 1 h. Protein bands were shown after 1-h exposure with GE’s Amersham ECL + Chemiluminescent CCD camera. The protein level was indicated as the value according to relative ratio to loading control GAPDH. 

### 2.11. Statistical Analysis

All data are presented as mean values ± standard deviation (S.D.). The differences were compared using the analysis of variance (ANOVA) among different groups. SPSS Statistical Package 20.0 (SPSS Inc., Chicago, IL, USA) was used to analyze these data. There was statistical significance of difference if *p* < 0.05.

## 3. Results

### 3.1. Establishment of NASH Model

NASH models were determined according to an earlier report [56]. The scores of all rats were more than 8 (Table 3). A NAFLD activity score (NAS) ≥5 was associated with a diagnosis of NASH, and <3 would be diagnosed as “not NASH” [56]. Therefore, the model was established successfully and would be useful in NASH experiment.

### 3.2. BP Increase Weight Loss of Rat NASH Model

There were statistical differences for body weight, liver index and epididymal fat weight between control and model groups after three months and ten days (Table 4, *p* < 0.05). Comparatively, *PPAR-α* silence increased the weight while blueberry juice and/or probiotics treatment reduced the weight compared to models (Table 4, *p* < 0.05).

### 3.3. BP Improve Antiinflammatory Activities of Rat NASH Model

There were statistical differences for the levels of inflammatory cytokines between control and model groups after three months and ten days (Table 5, *p* < 0.05). Comparatively, PPAR-α silence increased the levels while blueberry juice and/or probiotics treatment reduced the levels compared to models (Table 5, *p* < 0.05).

### 3.4. BP Improve Antioxidant Activities of Rat NASH Model

Serum biochemical index analysis showed that serum levels of ALT and AST were the highest in PIG compared to all other groups (Table 6) (*p* < 0.05). In contrast, the serum levels of SOD and GSH were lowest compared to all other groups (Table 6) (*p* < 0.05). Blueberry juice and/or BP reduced the serum levels of ALT and AST, and increased the levels of SOD and GSH in NASH models and the models with the treatment of PPAR-α inhibitor (Table 6) (*p* < 0.05). Serum levels of ALT and AST were lowest, and the levels of SOD and GSH were highest in CG (Table 6) (*p* < 0.05). Biochemical analysis showed that there was statistical significance of differences for the serum levels of SOD, GSH, ALT and AST among different groups (Table 6) (*p* < 0.05). The results suggest that BP improve antioxidant activities of rat NASH model.

### 3.5. Blueberry Juice and Probiotics Improve Lipid Patterns of Rat NASH Model

Lipid pattern analysis showed that serum MDA, TG, TC, and LDL-C reached the highest level in PIG compared to other groups (Table 7) (*p* < 0.05). In contrast, the serum HDL-C reached the lowest level. Blueberry juice and/or BP treatment reduced serum levels of MDA, TG, TC, and LDL-C, and increased the levels of HDL-C in NASH models and the models with the treatment of PPAR-α inhibitor. Comparatively, serum MDA, TG, TC, and LDL-C were at the lowest level and HDL-C was at the highest level in CG (Table 7) (*p* < 0.05). Lipid profile analysis also showed that there was statistical significance of differences for the serum levels of TG, TC, HDL-C, LDL-C and MDA among different groups (Table 7) (*p* < 0.05).

The results suggested that the combination of BP is better than only blueberry consumption for improving serum biochemical indices (Table 6) and lipid pattern (Table 7) in NASH models. When PPAR-α inhibitor was used, there was statistical significance of the differences between PIG and MG groups in most cases (*p* > 0.05) (Table 6 and Table 7), suggesting PPAR-α may play an important role in the progression of NASH. Blueberry juice and the combination of BP reduced the progression of NASH by improving its biochemical indices and lipid patterns in NASH models or the models with the treatment of PPAR-α inhibitor (*p* < 0.05) (Table 6 and Table 7). 

### 3.6. The Effects of PPAR-α Antagonist and/or Blueberry Juice on the Growth of Liver Cells

**Figure 2:**
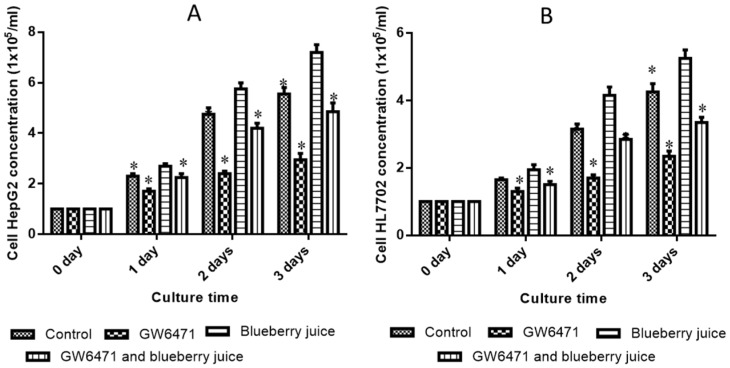
The effects of PPAR-α antagonist and blueberry juice on the growth of human liver cells: (**A**) the effects of PPAR-α antagonist and blueberry juice on the growth rate of cell line HL7702; and (**B**) the effects of PPAR-α antagonist and blueberry juice on the growth rate of liver cell line HepG2. All data are presented as mean value ± S.D. Five samples were analyzed in each group. * *p* < 0.05 via a blueberry juice group.

There was no statistical significance of differences for the cell concentrations among all groups in cell line HL7702 (*p* > 0.05) (Figure 2A) and cell line HepG2 (*p* > 0.05) (Figure 2B) before the treatment of PPAR α antagonist. After treatment, Figure 2A showed that GW6471 inhibited the growth rate of cell line HL7702 while blueberry juice increased the growth rates of HL7702 cells (*p* < 0.05). Meanwhile, blueberry reduced the inhibition of PPAR-α activity caused by GW6471 in HL7702 cells (*p* < 0.05). Similarly, Figure 2B shows that GW6471 inhibited the growth rate of cell line HepG2 while blueberry juice increased the growth rates of HepG2 cells (*p* < 0.05). Meanwhile, blueberry reduced the inhibition of PPAR-α activity caused by GW6471 in HepG2 cells (*p* < 0.05). All these results suggest that blueberry juice prevents the inhibition of PPAR-α activity caused by GW6471 in both kinds of liver cell lines.

### 3.7. Measurement of PPAR-α Activity

**Figure 3:**
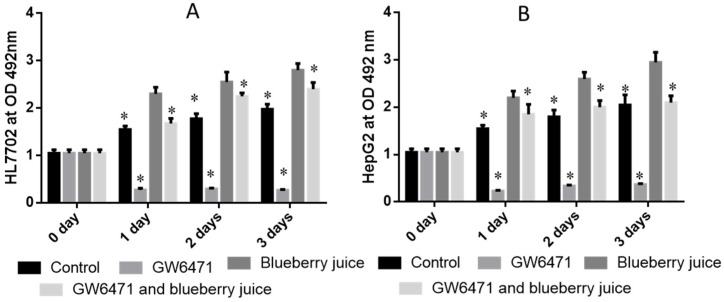
The activity of PPAR-α in different cells: (**A**) the effects of BP on the antagonist of PPAR-α in HL7702 cells; and (**B**) the effects of BP on the antagonist of PPAR-α in HepG2 cells. Five samples were analyzed in each group. All data are presented as mean value ± S.D. * *p* < 0.05 via a blueberry juice group BG.

There was no statistical significance of differences for the activity of PPAR-α among all groups in cell line HL7702 (*p* > 0.05) (Figure 3A) and cell line HepG2 (*p* > 0.05) (Figure 3B) before the treatment of PPAR-α antagonist. Compared with controls, the antagonist of PPAR-α reduced the activity of PPAR-α significantly in HL7702 (Figure 3A) and HepG2 (Figure 3B) liver cells (*p* < 0.05). There was statistical significance of differences in the PPAR-α activity between GW6471 and GW6471 + blueberry juice treatment in HL7702 (Figure 3A) and HepG2 (Figure 3B) cells (*p* < 0.05). 

**Figure 4:**
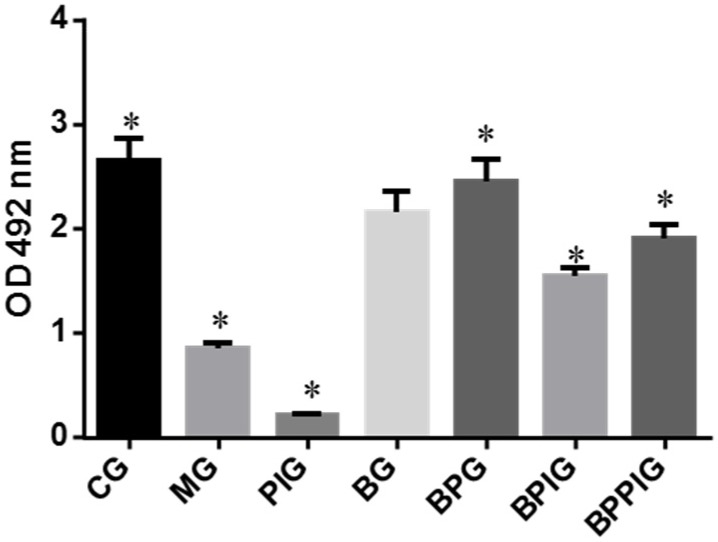
The activity of PPAR-α in different groups: The effects of BP on the antagonist of PPAR-α in different groups by using blood samples. Eight samples were analyzed in each group. All data are presented as mean value ± S.D. * *p* < 0.05 via a blueberry juice group BG.

Similarly, compared with controls, the antagonist reduced the activity of PPAR-α significantly in PIG (Figure 4, *p* < 0.05). There was statistical significance of differences in the PPAR-α activity between PIG and BPIG or BPPIG (Figure 4) (*p* < 0.05). All these results suggest that blueberry juice can prevent the inhibition of PPAR-α activity caused by GW6471.

### 3.8. Blueberry Juice and Probiotics Ameliorate NASH

**Figure 5:**
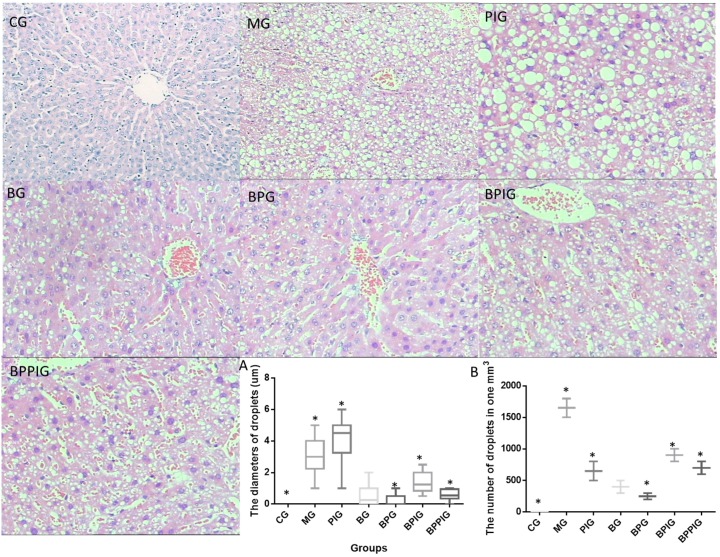
HE analysis for the effects of BP on NASH and liver damage: (**A**) the diameters of lipid droplets in different groups; and (**B**) the concentration of lipid droplets in different groups. Eight liver samples were analyzed in each group. All data are presented as mean value ± S.D. * *p* < 0.05 via a blueberry juice group BG.

Figure 5 showed the liver tissues stained by HE in different groups. For healthy rats, hepatic cells encircled a tubular section regularly, and all lipid droplets were small. In MG, the structures of hepatic cells were destructed and widely distributed with many lipid droplets. In PIG, the lobular structure was heavily destroyed and filled with many large lipid droplets compared to the liver tissues from the model group. Many large lipid droplets were produced when the activity of PPAR-α was blocked by its antagonist GW6471, suggesting that PPAR-α plays an important role in maintaining the normal structure of liver cells. The size of lipid droplets was decreased in the BG and BPG groups compared to the tissues in MG, PIG, BPIG, and BPPIG groups. In BPG, the hepatic cords were radially arranged around a central vein and there were fewer lipid droplets. In the BPPIG, the hepatic structure was destroyed and the lipid droplets were small. Although the number of droplets in BPIG was greater than in PIG (Figure 5A), the average size of droplets was larger in PIG than in BPIG (Figure 5B). These findings suggested that the combination of blueberry juice and probiotics attenuates NASH better than the group only fed blueberry juice.

**Figure 6:**
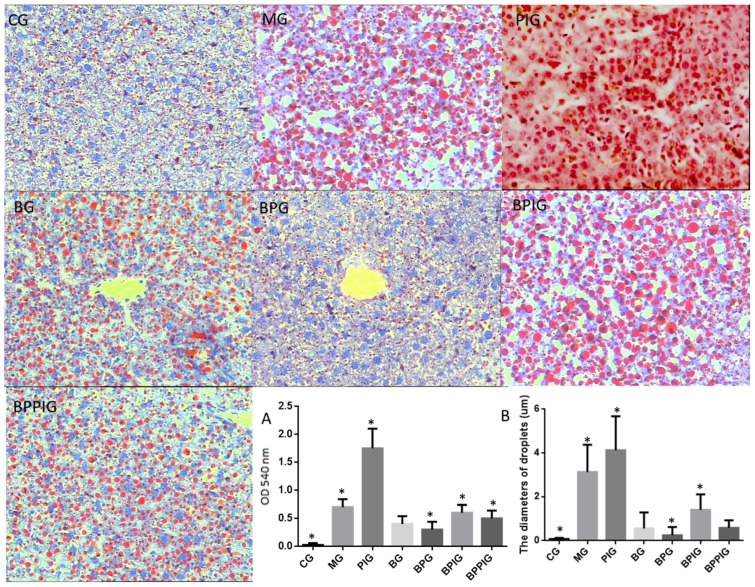
ORO analysis for the effects of BP on NASH and liver damage: (**A**) the absorbing value of the solvent of ORO extraction at 540 nm in different groups; and (**B**) the diameters of droplets in different groups. Eight liver samples were analyzed in each group. All data are presented as mean value ± S.D. * *p* < 0.05 via a blueberry juice group BG.

Figure 6 shows the liver tissues stained using ORO from different groups. In CG, the liver tissues were little stained with ORO while the liver tissues were most stained with ORO in PIG. Blueberry juice reduced the staining in animal models and the model treated with GW6471. BP treatment reduced the staining better than only blueberry juice. The absorbing values for ORO showed a similar changing trend in different groups (Figure 6A). The value was highest in PIG and lowest in CG. BP reduced the values in BG, BPG, BPIG and BPPIG. Comparatively, the number and size of droplets were highest in PIG compared to other groups while the number and size of droplets were lowest in CG compared to other groups (Figure 6B). Blueberry juice and/or probiotics treatment reduced the number and size of droplets compared to PIG (Figure 6B, *p* < 0.05).

### 3.9. Liver Apoptosis

**Figure 7:**
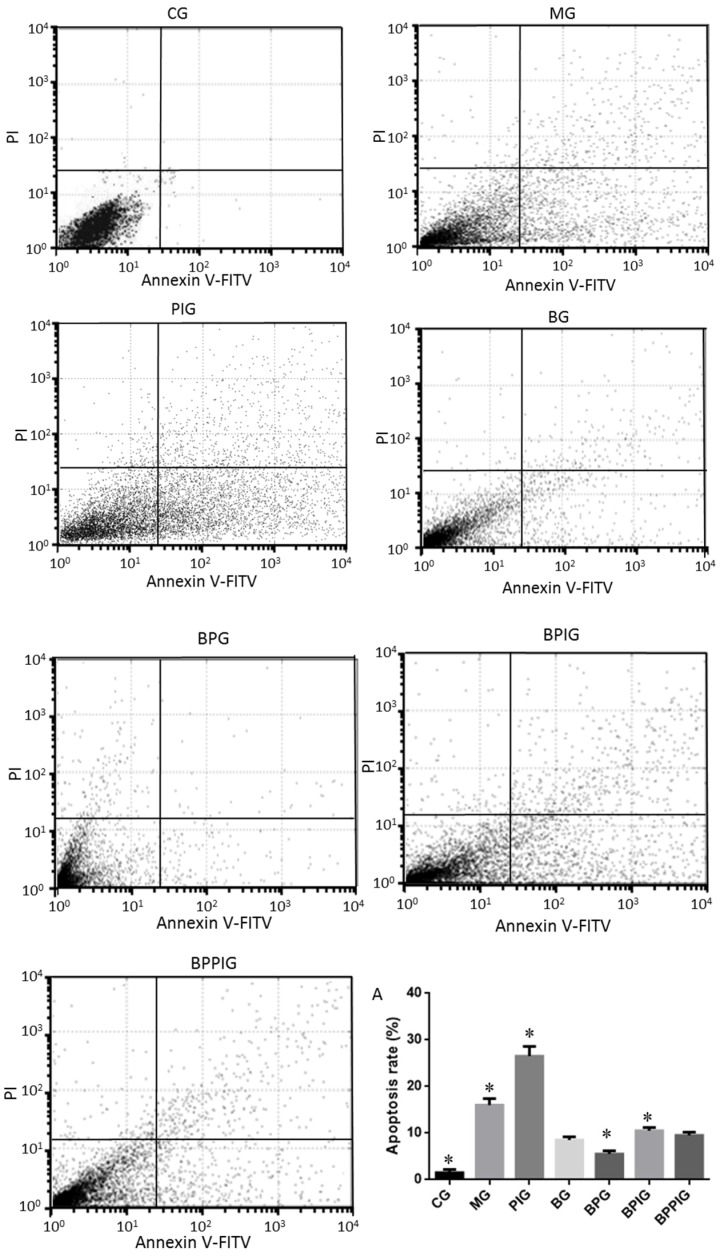
Flow cytometer analysis for the apoptosis of liver tissue: apoptotic rate of liver tissues in different groups (**A**). Eight liver samples were analyzed in each group. All data are presented as mean value ± S.D. * *p* < 0.05 via a blueberry juice group BG.

The degree of apoptosis for NASH liver tissues was analyzed by flow cytometer. The results showed that the apoptotic rate was lowest in CG and highest in PIG compared to other groups (Figure 7) (*p* < 0.05). Blueberry juice treatment and/or BP treatment reduced apoptotic rates in NASH models. Blueberry juice treatment and/or BP treatment also significantly reduced apoptotic rates in NASH models treated with GW6471. All these results showed that of BP treatment reduced apoptotic rates of NASH than only blueberry consumption (Figure 7A).

### 3.10. Relative mRNA Levels of PPAR-α, SREBP-1c and PNPLA-3

**Figure 8:**
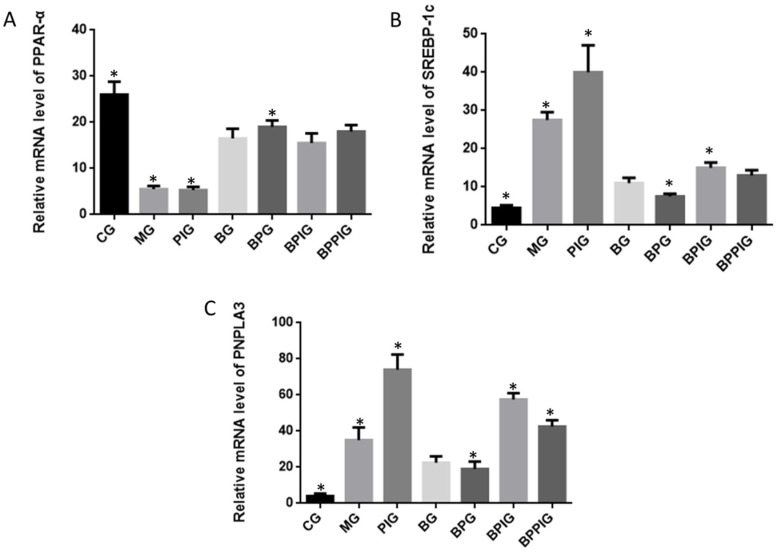
Real-time quantitative RT-PCR analysis of the mRNA levels of SREBP-1c/PNPLA-3 pathway and its interacting molecules. (**A**) Relative mRNA level of PPAR-α; (**B**) Relative mRNA level of SREBP-1C; (**C**) Relative mRNA level of PNPLA3. Eight liver samples were analyzed in each group. All data are presented as mean value ± S.D. * *p* < 0.05 via a blueberry juice group BG.

qRT-PCR analysis indicated that the mRNA levels of Srebp-1c and Pnpla-3 were higher in MG, PIG, BPIG and BPPIG than in CG, BG, and BPG (Figure 8) (*p* < 0.05). Comparatively, the mRNA level of *PPAR-α* was higher in CG, BG, BPG, BPIG, and BPPIG than in MG and PIG (*p* < 0.05) (Figure 8). The results suggest that BP increased the mRNA levels of PPAR-α, which reduced the level of SREBP-1c and PNPLA-3. After the treatment of PPAR-α inhibitor, the mRNA level of PPAR-αwas stable but the mRNA levels of SREBP-1c and PNPLA-3 were reduced, suggesting that PPAR-αantagonist reduced the activity of PPAR-α and resulted in the increase of the mRNA levels of SREBP-1c and PNPLA-3. Thus, the results confirmed that blueberry juice can inhibit the progression of NASH by down-regulating SREBP-1c/PNPLA-3 pathway via PPAR-α.

### 3.11. Relative mRNA Levels of Apoptosis-Inducing Factors (caspase-3 and Bcl-2)

**Figure 9:**
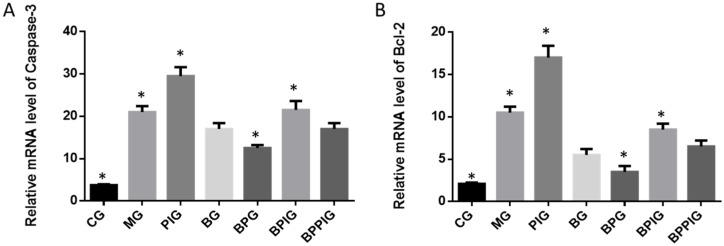
Real-time quantitative RT-PCR analysis of the mRNA levels of apoptotic factors: (**A**) relative mRNA levels of caspase-3; and (**B**) relative mRNA levels of Bcl-2. Five liver samples were analyzed in each group. All data are presented as mean value ± S.D. * *p* < 0.05 via a blueberry juice group BG.

qRT-PCR analysis indicated that the mRNA levels of caspase-3 and Bcl-2 were higher in MG, PIG, BPIG and BPPIG than in CG, BG, and BPG (Figure 9) (*p* < 0.05). The results suggest that BP treatment reduced the mRNA level of caspase-3 and Bcl-2. After the treatment of PPAR-α inhibitor, the mRNA level of PPAR-α was stable but the mRNA levels of caspase-3 and Bcl-2 were increased, suggesting that the inhibition of PPAR-α activity will increase the mRNA levels of caspase-3 and Bcl-2. 

### 3.12. Relative mRNA Levels of Inflammatory Cytokines (IL-6 and TNF-α)

**Figure 10:**
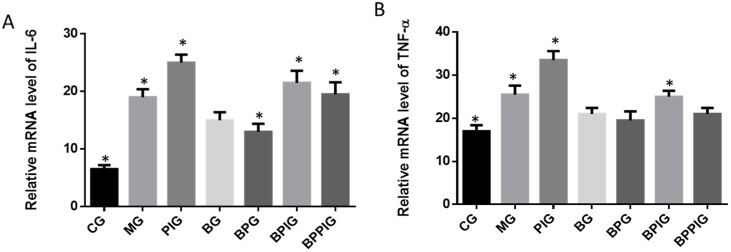
Real-time quantitative RT-PCR analysis of the mRNA levels of inflammatory cytokines: (**A**) relative mRNA levels of IL-6; and (**B**) relative mRNA levels of TNF-α. Five liver samples were analyzed in each group. All data are presented as mean value ± S.D. * *p* < 0.05 via a blueberry juice group BG.

Similar to caspase-3 and Bcl-2, qRT-PCR analysis indicated that the mRNA levels of IL-6 and TNF-α were higher in MG, PIG, BPIG and BPPIG than in CG, BG, and BPG (Figure 10) (*p* < 0.05). The results suggest that BP treatment will reduce the level of IL-6 and TNF-α. After the treatment of PPAR-α inhibitor, the mRNA level of PPAR*-*α was stable but the mRNA levels of IL-6 and TNF-α were increased, suggesting that the inhibition of PPAR-α activity will result in the increase of the mRNA levels of IL-6 and TNF-α. 

### 3.13. Relative Protein Levels of PPAR-α, SREBP-1c and PNPLA-3

**Figure 11:**
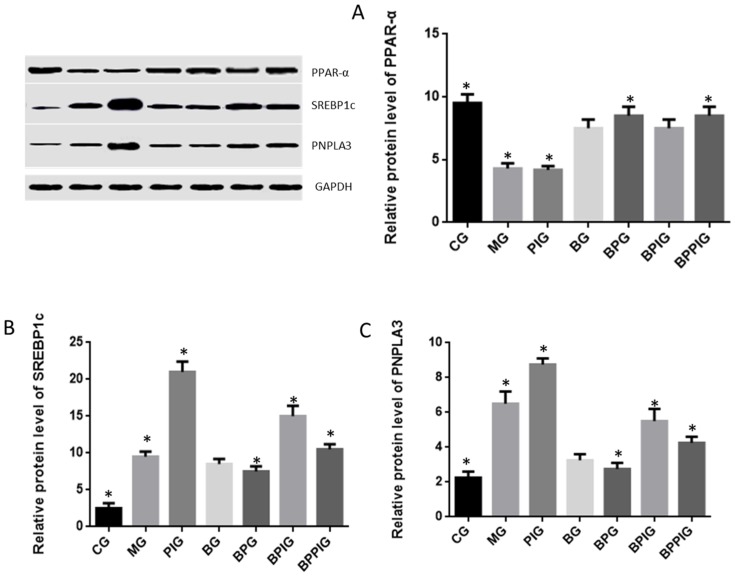
Western blot analysis of the protein levels of SREBP-1c/PNPLA-3 pathway and its interacting molecules. (**A**) relative protein level of PPAR-α; (**B**) relative protein level of SREBP-1c; (**C**) relative protein level of PNPLA3. Eight liver samples were analyzed in each group. All data are presented as mean value ± S.D. * *p* < 0.05 via a blueberry juice group BG.

qRT-PCR analysis indicated that the protein levels of SREBP-1c and PNPLA-3 were higher in MG, PIG, BPIG and BPPIG than in CG, BG, and BPG (Figure 11) (*p* < 0.05). Comparatively, the protein level of PPAR-α was higher in the CG, BG, BPG, BPIG, and BPPIGs than in the MG and PIG groups (*p* < 0.05) (Figure 11). The results suggest that BP increased the protein levels of PPAR-α while the inhibition of PPAR-α activity will increase the level of SREBP-1c and PNPLA-3. Thus, the results confirmed that blueberry juice can inhibit the progression of NASH by affecting SREBP-1c-PNPLA-3 pathway via PPAR-α.

### 3.14. Relative Protein Levels of Apoptosis-Inducing Factors (caspase-3 and Bcl-2)

**Figure 12:**
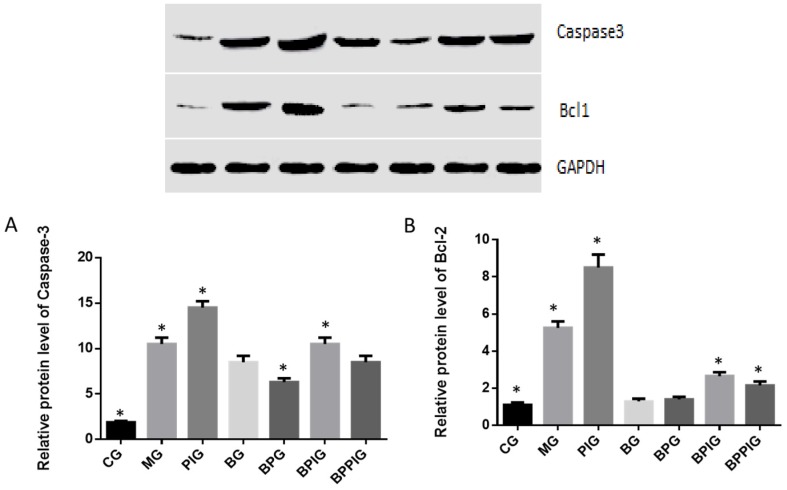
Western blot analysis of the protein levels of casepase-3 and Bcl-2. Eight liver samples were analyzed in each group. (**A**) relative protein level of caspase-3; (**B**) relative protein level of Bcl-2. All data are presented as mean value ± S.D. * *p* < 0.05 via a blueberry juice group BG.

qRT-PCR analysis indicated that the protein levels of caspase-3 and Bcl-2 were higher in MG, PIG, BPIG and BPPIG than in CG, BG, and BPG (Figure 12) (*p* < 0.05). The results suggest that BP reduces the protein level of caspase-3 and Bcl-2. After the treatment of PPAR-α inhibitor, the protein level of PPAR-α was stable but the protein levels of casepase-3 and Bcl-2 were increased, suggesting that PPAR-α antagonist increased the protein levels of caspase-3 and Bcl-2.

### 3.15. Relative Protein Levels of Inflammatory Cytokines (IL-6 and TNF-α)

**Figure 13:**
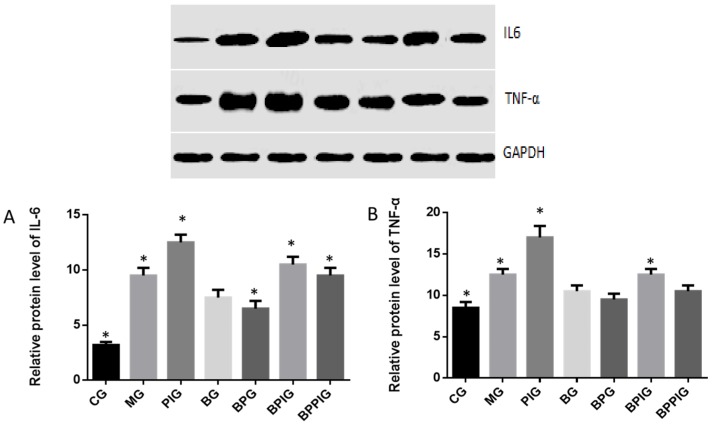
Western blot analysis of the protein levels of IL-6 and TNF-α. Eight liver samples were analyzed in each group. **A**, relative protein level of IL-6. **B**, relative protein level of TNF-α. All data are presented as mean value ± S.D. * *p* < 0.05 via a blueberry juice group BG.

Just as caspase-3 and Bcl-2, Western Blot analysis indicated that the protein levels of IL-6 and TNF-α were higher in MG, PIG, BPIG and BPPIG than in CG, BG, and BPG (Figure 13) (*p* < 0.05). The results suggest that BP increased the protein levels of PPAR-α, which reduced the level of IL-6 and TNF-α. After the treatment of PPAR-α inhibitor, the protein level of PPAR-α was stable but the protein levels of IL-6 and TNF-α were increased, suggesting that PPAR-α antagonist increased the protein levels of IL-6 and TNF-α. 

## 4. Discussion

Inflammation is the main reason for the pathogenesis of NASH [24,25]. Quantitative real-time PCR and Western blot analysis showed that the combination of blueberry juice and probiotic bacteria had a significant inhibitory effect on inflammatory cytokines IL-6 and TNF-α. The levels of inflammatory cytokines in the BPG group was lower than that in the BG group (*p* < 0.05) (Figure 10 and Figure 13), suggesting that there is a cumulative effect of the combination of BP for NASH treatment by reducing the levels of cytokines. On the other hand, the combination of BP also increased the level of PPAR-α. PPAR-α has been reported to play an important role in many physiological activities, such as lipid metabolism [62,63], glucose homeostasis [64] and anti-inflammatory action [65,66,67]. PPAR-α has been reported to play an important role in many physiological activities, such as lipid metabolism [62,63], glucose homeostasis [64] and anti-inflammatory action [65,66]. At cell levels, blueberry increased the growth rate of HL7702 (Figure 3A) and HepG2 (Figure 3B) cells compared to other groups without blueberry treatment (*p* < 0.05). Similarly, compared with controls, the antagonist reduced the activity of PPAR-α significantly in PIG (Figure 4, *p* < 0.05). All these results suggest that blueberry juice may reduce the inhibitory functions of the antagonist for PPAR-α and promote the growth of liver cells. However, the exact molecular mechanism remains unknown. Further work is needed to confirm the results and explore the related molecular mechanism.

The dysfunction of lipid metabolism is another main reason for the pathogenesis of NASH. The dysfunction of lipid metabolism often produces reactive oxygen species (ROS), which lead to hepatic apoptosis. On the other hand, caspase-3 and Bcl-2 are important apoptotic biomarkers. Blueberry and/or probiotics consumption increased the levels of SOD and GSH, and reduced the levels of ALT, AST, caspase-3 and Bcl-2. Thus, the results will prevent ROS production and apoptosis in hepatocytes and maintain the normal development and homeostasis of liver tissues. This study reports the pathogenesis of NASH and shows the destruction of hepatic tissues because of the changes in the activity of PPAR-α. According to an earlier report, the consumption of *n*-3 long-chain polyunsaturated fatty acids reduced the ratios of SREBP-1c to PPAR-α, which contributes to favored fatty acid oxidation and attenuate steatosis [68]. The combination of blueberry juice and probiotic bacteria increased the level of PPAR-α and reduced the level of SREBP-1c. According to an earlier report, oxidative stresses increase in NASH patients by increasing total oxidant status. Antioxidant treatment and increasing total antioxidant status are therapeutic options for NASH patients [69]. Elevated serum level of ALT is a marker of inflammation and oxidative stress [70]. AST is also related to oxidative stress and increasing all-cause mortality [71]. SOD is an important antioxidant biomarker by detoxifying normally generated reactive oxygen species. The increase of PPAR-α will enhance antioxidant functions of liver cells by affecting the level of SOD, ALT and AST [13,72]. In contrast, SREBP-1c will increase the level of ROS in hepatocytes and aggravate inflammatory injury of liver tissues [73]. Therefore, the results reduced lipid deposition, inflammation, necrosis, and fibrosis in hepatic tissues by enhancing the activity of the anti-oxidant enzyme SOD and decreasing the levels of biomarkers of oxidative stress ALT and AST. Comparatively, the antioxidant activities were measured by earlier reports. There was positive correlation between antioxidant activity and the polyphenols and vitamin C content in *Actinidia kolomikta*, *Actinidia arguta*, and *Actinidia chinensis* [74]. Another report showed that strawberry extract had no cytotoxic effects on HepG2 cell lines with high concentration of strawberry extracts, while 250 µg/mL had cytotoxic for HepG2 after 48- and 72-hour culture [75].

Hepatocyte apoptosis is greatly enhanced in NASH patients with NASH and the apoptosis is associated with NASH severity, suggesting that anti-apoptotic treatment will be useful for preventing the progression of NASH [76]. Flow cytometer analysis showed that the combined therapy of BP had a significant inhibitory effect on hepatic apoptosis. The apoptotic rate in the BPG group was lower than that in the BG group (*p* < 0.05) (Figure 7A), also suggesting that there was a synergistic effect of the combination of BP for NASH treatment. However, an exact molecular mechanism for the synergetic functions of BP still needs to be explored. 

According to earlier reports, SREBP-1c plays a critical role in NASH progression and involves in tissue apoptosis [3,77], cell cycle control [78,79], transcription regulation [80], lipid metabolism [81,82], and many other life activities. All these are related to the development of NASH. Polyphenol is one of main component of blueberry juice [83,84] and observed to reduce the SREBP-1c level [85]. Present findings indicated that BP increase PPAR-α activity, which prevented the expression of SREBP-1c and resulted in the decrease of PNPLA-3 level.

Furthermore, BP can reduce the severity of NASH even antagonist of PPAR-α is used. The result suggests that other molecular mechanisms maybe existed for controlling NASH progression via BP. Probiotics and blueberry cooperatively increase the protecting functions for liver tissues of NASH models. The probiotics mixture with *Bifidobacterium lactis*, *Lactobacillus bulgaricus* and *Streptococcus thermophiles* had an additional effect to prevent NASH and/or its related events. The reason is complex. Systemic and local inflammation of NASH is often associated with the resident microbiota of the human gastro-intestinal tract. Thus, the administration of probiotics mixture may modulate systemic immune, and prevent the metabolic syndrome, liver injury, inflammatory bowel disorder, and enteritis [86]. All these activities may be beneficial for improving NASH. 

Clearer molecular mechanism still needs to be explored in the future. On the other hand, PPAR-α activation prevents triglyceride synthesis in liver cells, which may be caused by inhibiting SREBP1 activity [87] while SREBP-1c expression has been widely reported to be associated with steatosis [68,88]. PNPLA-3 is associated with lipid accumulation in hepatic tissues, especially in steatosis progression. Steatosis progression will contribute to increasing the risk of NASH [89]. One important question should be considered here. SREBP-1c is not involved in cholesterol homeostasis. Why were the cholesterol levels changed by the NASH diet and PPARα antagonist? According to previous reports, PPAR-α and PPAR-γ activation induce cholesterol removal from human cells [90]. This result suggests that PPARα antagonist will cause the increase of cholesterol levels. On the other hand, cholesterol overloading will result in the increase in the expression of SREBP-1c [91]. BP can increase PPAR-α level, resulting in the decrease of cholesterol levels.

SIRT1 regulates lipid homeostasis by upregulating PPAR-α [92]. Present results showed the increased level of PPAR-α would reduce the levels of SREBP-1c and PNPLA-3. Thus, PPAR-α may inhibit the activity of SREBP-1c and PNPLA-3 ways. Thus, SIRT-1 signaling pathway may control SREBP-1c/PNPLA-3 pathway. 

There were the following limitations in the present work: (1) The antagonist of PPAR-α was not used in healthy animals. The following reasons were considered: present work mainly focuses on animal NASH models, and, to reduce the number of animals and injury, fewer animals were used finally; (2) A clinical trial was not performed, although all the materials are safe for human consumption. Therefore, further work is needed to be done in NASH patients in the future; (3) Although probiotic has been regarded as a treatment option for NASH because of its effects on the gut flora, no randomized clinical trial was identified for such proposal [93]. We guess probiotic alone cannot prevent the progression of NASH. Therefore, the experiment was not performed here; (4) SREBP1c-PNPLA3 pathway has been regarded as a “disease module” that promotes hepatic fibrogenesis [94] while the latter is associated with the progression of NASH. Thus, we guess PPAR α may affect NASH development by affecting SREBP1c-PNPLA3 pathway. Further work is needed to confirm this proposal.

## 5. Conclusions

This study showed that BP has synergistic effects for preventing the development of NASH. BP can reduce the injuries and apoptosis of liver tissues in NASH models by improving the activity of PPAR-α, which inhibits the levels of SREBP-1c and PNPLA-3. The results increased the levels of anti-oxidant enzymes to remove oxygen free radicals, reduced lipid peroxidation and protected hepatocytes. Therefore, BP reduces the apoptosis and inflammation of hepatocytes in NASH by affecting the SREBP-1c/PNPLA-3 pathway via PPAR-α. BP should be developed food additives for preventing the progression of NASH.

## Figures and Tables

**Table 1:** Ingredient and nutrient composition of the diets (g/kg).

Ingredients (g/kg)	Normal Diet	HFD Diet
Casein	200.0	200.0
Starch	615.0	435.0
Sucrose	-	150.0
Corn oil	80.0	-
lard oil	-	100.0
cholesterol	-	20
Cellulose	50.0	50.0
Vitamin-Mineral mixture	50.0	50.0
dl-Methionine	3.0	3.0
Choline chloride	2.0	2.0
Chromium, mg/kg	0.066	0.097

**Table 2:** The primer sequences used in qRT-PCR.

		Sequences (5′-3′)	Size (bp)
PPAR-α	Forward	GTCAGCTGCCCTGCTGTCCC	130
	Reverse	CGAAAGAAGCCCTTGCAGCC	
SREBP-1c	Forward	CGCTTCTTACAGCACAGCAA	220
	Reverse	TGCCCAAGGACAAGGGGCTA	
PNPLA-3	Forward	CGCACTTTCTTCGGCTGCTC	150
	Reverse	AATGTTGAAGAACGGGTGGA	
IL-6	Forward	CACAACAGACCAGTATATAC	160
	Reverse	GTATTTCTGGAAGTTTCAG	
TNF-α	Forward	GTGGCGGGGGCCACCACGCTC	141
	Reverse	CGAGTTTTGAGAAGATGATC	
Caspase3	Forward	ATGTCAGCTCGCAATGGTAC	210
	Reverse	CGTTCCAAAAATTACTCC	
Bcl-2	Forward	GTGCACCGAGACACGGCTGC	140
	Reverse	CGACGGTAGCGACGAGAGAA	
GAPDH	Forward	TGTTCCAGTATGACTCTACC	130
	Reverse	TCACCCCATTTGATGTTAGC	

**Table 3:** NASH model evaluated by NASH Research Network Scoring System Definitions and Scores in Study Set.

Items	Definition	Scores	*n* = 48
Steatosis Grade	Low- to medium-power evaluation of parenchymal involvement by steatosis		
	<5%	0	0%
	5%–33%	1	0%
	>33%–66%	2	29%
	>66%	3	70%
Location	Predominant distribution pattern		
	Zone 3	0	0%
	Zone 1	1	2%
	Azonal	2	56%
	Panacinar	3	42%
Microvesicular steatosis	Contiguous patches		
	Not present	0	83%
	Present	1	17%
Fibrosis stage			
	None	0	42%
	Perisinusoidal or periportal	1	2%
	Mild, zone 3, perisinusoidal	1	2%
	Moderate, zone 3, perisinusoidal	1	8%
	Portal/periportal	1	8%
	Perisinusoidal and portal/periportal	2	25%
	Bridging fibrosis	3	4%
	Cirrhosis	4	4%
Inflammation			
Lobular inflammation	Overall assessment of all inflammatory foci		
	No foci	0	0%
	<2 foci per 200× field	1	2%
	2–4 foci per 200× field	2	56%
	>4 foci per 200× field	3	42%
Microgranulomas	Small aggregates of macrophages		
	Absent	0	63%
	Present	1	37%
Large lipogranulomas	Usually in portal areas or adjacent to central veins		
	Absent	0	73%
	Present	1	27%
Portal inflammation	Assessed from low magnification		
	None to minimal	0	52%
	Greater than minimal	1	48%
Liver cell injury			
Ballooning			
	None	0	21%
	Few balloon cells	1	58%
	Many cells/prominent ballooning	2	21%
Acidophil bodies			
	None to rare	0	87%
	Many	1	13%
Pigmented macrophages			
	None to rare	0	83%
	Many	1	17%
Megamitochondria			
	None to rare	0	75%
	Many	1	24%
Other findings			
Mallory’s hyaline	Visible on routine stains		
	None to rare	0	67%
	Many	1	33%
Glycogenated nuclei	Contiguous patches		
	None to rare	0	52%
	Many	1	48%
Diagnostic classification			
Not steatohepatitis		0	0%
Possible/borderline		1	0%
Definite steatohepatitis		2	100%

**Table 4:** The comparison of liver and fat body weights among different groups.

	CG	MG	PIG	BG	BPG	BPIG	BPPIG
Body weight (g)	368.2 ± 32.7	397.4 ± 36.6	410.4 ± 47.5	390.3 ± 32.6	384.8 ± 28.9	394.7 ± 36.8	388.4 ± 33.2
Liver index (%)	2.6 ± 0.3	3.2 ± 0.4	3.4 ± 0.5	3.0 ± 0.3	2.8 ± 0.3	3.1 ± 0.3	2.9 ± 0.3
epididymal fat weight (g)	8.4 ± 1.3	9.8 ± 1.6	11.2 ± 1.8	9.1 ± 1.6	8.8 ± 1.3	9.3 ± 1.4	8.9 ± 1.5

Note: Liver index was presented as liver weight/body weight × 100%. All rat NASH models were evenly and randomly assigned to 6 groups: model group (MG, the model rats were intraperitoneally injected with 50 μL/kg saline solution and orally received 20 mL/kg liquid placebo daily); blueberry juice group (BG, the model rats were intraperitoneally injected with 50 μL/kg saline solution and orally received 10 mL/kg blueberry juice and 10 mL/kg liquid placebo daily); Blueberry juice and probiotic bacteria group (BPG, the model rats were intraperitoneally injected with 50 μL/kg normal saline solution and orally received 10 mL/kg blueberry juice and 10 mL/kg probiotics daily); PPAR-α inhibitor group (PIG, the model rats were intraperitoneally injected with 50 μL/kg PPAR-α in saline solution); blueberry juice and PPAR-α inhibitor group (BPIG, the model rats were intraperitoneally injected with 50 μL/kg PPAR-α in saline solution and orally received 10 mL/kg blueberry juice and 10 mL/kg liquid placebo daily); and blueberry juice, probiotics, and PPAR-α inhibitor group (BPPIG, the model rats were intraperitoneally injected with 50 μL/kg PPAR-α in saline solution and orally received 10 mL/kg blueberry juice and 10 mL/kg probiotics daily). Meanwhile, 8 healthy rats were used as a control group (CG, the healthy rats were intraperitoneally injected with 50 μL/kg normal saline solution and orally received 20 mL/kg liquid placebo daily).

**Table 5:** The comparison of inflammatory cytokines among different groups.

	CG	MG	PIG	BG	BPG	BPIG	BPPIG
IL-6 (pg/mL)	12.34 ± 2.65	42.59 ± 6.18	46.81 ± 7.52	28.42 ± 5.17	22.32 ± 4.77	38.52 ± 5.34	29.33 ± 4.18
TNF α (pg/mL)	5.42 ± 2.41	14.59 ± 12.03	16.36 ± 2.87	12.35 ± 2.44	9.34 ± 1.38	13.55 ± 2.69	11.46 ± 2.79

**Table 6:** Biochemical parameters of enzyme activities for NASH.

Group (*n* = 8)	SOD (U/mL)	GSH (ng/L)	ALT (U/mL)	AST (U/mL)
CG	27.24 ± 3.26 ^b,^^c,d,f^	24.15 ± 2.14 ^b,^^d,f^	45.12 ± 10.43 ^b,^^c,d,e^	103.32 ± 25.17 ^b,^^c,f^
MG	12.25 ± 4.16 ^a,c,d,e,f^	13.23 ± 1.93 ^a,^^c,^^e,f^	87.79 ± 8.40 ^a,^^c,^^e,^	208.26 ± 19.64 ^a,^^c,^^e^
PIG	9.34 ± 2.32 ^a,^^b,^^c,d,e,f^	10.28 ± 1.74 ^a,^^b,^^c,e,f^	123.79 ± 12.36 ^a,^^b,^^c,e^	289.42 ± 24.48 ^a,^^b,^^c,e^
BG	22.34 ± 3.48 ^a,^^b,^^d,e^	22.38 ± 1.02 ^b,^^a,d,f^	61.45 ± 12.18 ^b,^^a,d,^	146.19 ± 25.28 ^b,^^d,f^
BPIG	17.68 ± 2.43 ^a,^^b,^^c,e^	14.28 ± 1.61 ^a,^^c,^^e^	105.25 ± 15.82 ^a,^^b,^^c,e^	253.24 ± 17.54 ^a,^^b,^^c,e,f^
BPG	28.52 ± 4.53 ^b,^^c,d,f^	25.64 ± 2.28 ^b,^^c,d,f^	56.34 ± 12.16 ^b,^^a,d^	128.63 ± 22.38 ^b,^^d,f^
BPPIG	20.25 ± 4.47 ^a,^^b,^^e^	17.331 ± 1.64 ^a,^^b,^^c,d,e^	95.18 ± 7.28 ^a,^^c,^^e^	186.61 ± 22.35 ^a,^^c,^^d,e^

Note: Eight blood samples were analyzed in each group. All data are presented as mean value ± S.D. ^a^ *p* < 0.05 vs. the control group (CG); ^b^ *p* < 0.05 vs. the model group (MG); ^c^ *p* < 0.01 vs. the BG group; ^d^ *p* < 0.05 vs. BPIG (Blueberry juice and the treatment of PPAR-α inhibitor); ^e^ *p* < 0.05 vs. the BPG (BP treatment); ^f^ *p* < 0.05 vs. the BPPIG group (BP and the treatment of PPAR-α inhibitor).

**Table 7:** Biochemical parameters of lipid metabolism for NASH (mmol/L).

Group (*n* = 8)	TG	TC	HDL-C	LDL-C	MDA
CG	0.84 ± 0.16 ^b,^^c,d,f^	2.55 ± 0.49 ^b,^^f^	1.36 ± 0.28 ^b,^^c,d,f^	1.13 ± 0.48 ^b,^^c,d,e,f^	0.48 ± 0.17 ^b,^^d,f^
MG	1.76 ± 0.42 ^a,c,d,e,f^	3.34 ± 1.50 ^a,^^c,^^d,e,f^	0.67 ± 0.48 ^a,^^c,^^e,f^	1.84 ± 0.66 ^a,c,e,f^	1.45 ± 0.44 ^a,^^c,^^d,e,f^
PIG	2.38 ± 0.32 ^a,^^b,^^c,d,e,f^	3.64 ± 1.29 ^a,^^b,^^c,d, e,f^	0.56 ± 0.29 ^a,^^b,^^c,e,f^	2.09 ± 0.43 ^a,^^b,^^c,e,f^	1.64 ± 0.37 ^a,^^b,^^c,d,e,f^
BG	1.19 ± 0.15 ^a,^^b,^^d,e,f^	3.26 ± 0.74 ^b,^^d,f^	1.12 ± 0.29 ^a,^^b,^^d^	1.52 ± 0.68 ^a,^^b,^^d,e,^	0.56 ± 0.39 ^b,^^d,f^
BPIG	2.03 ± 0.65 ^a,^^b,^^c,e,f^	3.47 ± 0.53 ^a,^^c,^^e,f^	0.76 ± 0.25 ^a,^^c,^^e,f^	1.73 ± 0.28 ^a,^^c,^^e^	1.12 ± 0.36 ^a,^^b,^^c,e,f^
BPG	0.92 ± 0.31 ^a,^^b,^^c,d,f^	3.12 ± 0.46 ^b,^^d,f^	1.24 ± 0.30 ^b,^^d,f^	1.36 ± 0.59 ^a,^^b,^^c,d,f^	0.41 ± 0.28 ^b,^^a,d,f^
BPPIG	1.26 ± 0.18 ^a,^^b,^^c,d,e^	3.52 ± 0.68 ^a,^^b,^^c,d,e^	0.98 ± 0.27 ^a,^^b,^^c,d,e^	1.66 ± 0.49 ^a,^^b,^^e^	0.72 ± 0.38 ^a,^^b,^^c,d,e^

Note: Eight blood samples were analyzed in each group. All data are presented as mean value ± S.D. ^a^ *p* < 0.05 vs. the control group (CG); ^b^ *p* < 0.05 vs. the model group (MG); ^c^ *p* < 0.01 vs. the BG group; ^d^ *p* < 0.05 vs. BPIG (Blueberry juice and the treatment of PPAR-α inhibitor); ^e^ *p* < 0.05 vs. the BPG (Blueberry juice and probiotics); ^f^ *p* < 0.05 vs. the BPPIG group (Blueberry juice and probiotics and the treatment of PPAR-α inhibitor).
